# Supplementary material for: Seroprevalence of anti‐SARS‐CoV‐2 antibodies in COVID‐19 patients and healthy volunteers up to 6 months post disease onset
Source: Eur J Immunol. 2020 Nov 10;50(12):2025–40. doi: 10.1002/eji.202048970 (PMC7756220; doi:10.1002/eji.202048970)
Supplement: Supplementary file 1 — Supporting Information [file EJI-50-2025-s001.pdf]

# Suppl Figure 1

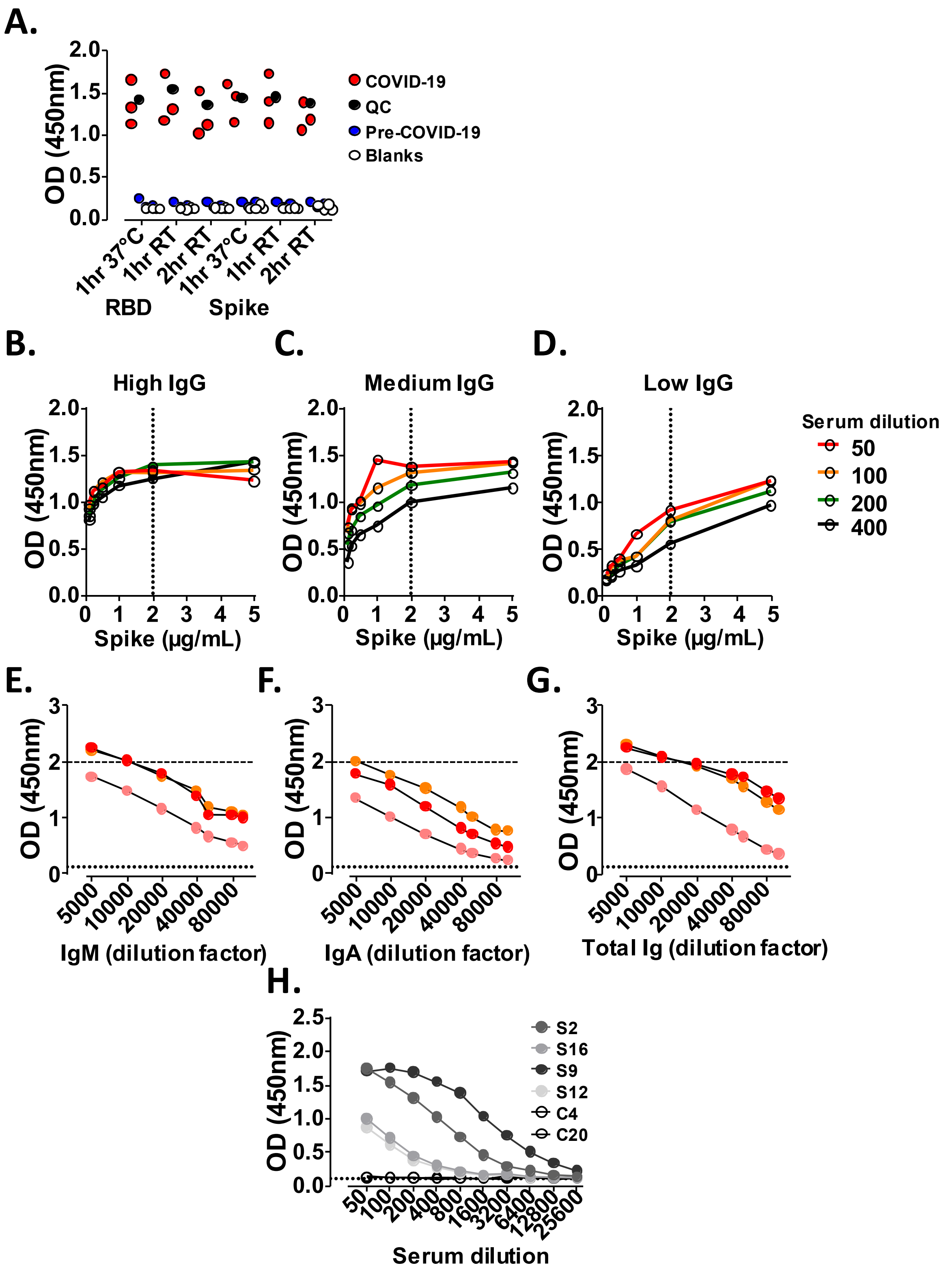


**Supplementary Figure 1. SARS-CoV-2 ELISA setup**. SARS-CoV-2 IgG antibody detection in serum samples from SARS-CoV-2 PCR-positive subjects or pre-COVID-19 controls using Immulon 4HBX 96-well plates coated with SARS-CoV-2 protein. Absorbance (Optical density- OD) was evaluated at 450nm at Tecan infinite M200 reader. **A**) RBD or Spike protein were coated at 2µg/ml, three (red) or QC control (black) COVID-19 serum and three pre-COVID-19 samples were loaded for indicated time and at indicated temperature. **B-D**) Representative example of serum samples of B) high, C) medium and D) low titre were assessed at indicated dilutions on wells coated with 0.5, 1, 2 and 5µg/ml concentrations of Spike protein. **E-G**) Secondary antibody dilution titration, E) IgM, F) IgA, and G) total Ig at indicated dilution on 96-well plate coated with 2µg/ml RBD protein. Dashed line indicates blank values. **H**) Serum dilution example of plates coated with 2µg/ml RBD protein detecting IgG in four COVID-19 samples (S) and two pre-COVID-19 samples (C).

# Suppl Figure 2

## A.

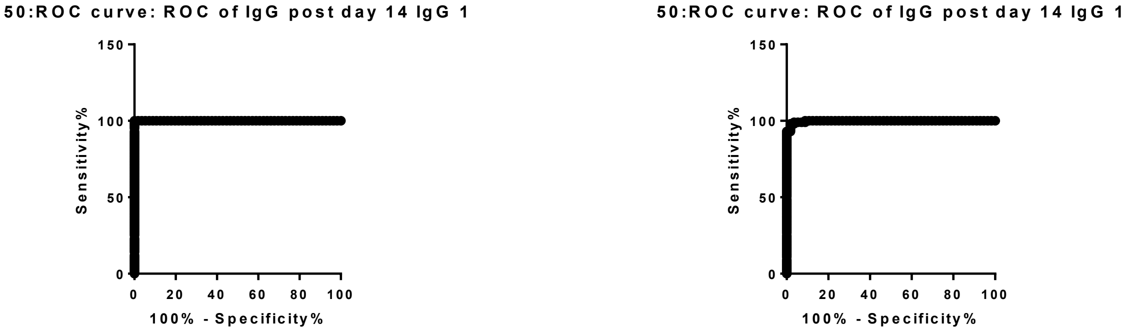


**RBD**

| Cut off | Sensitivity % | CI (%) | Specificity % | CI (%) |
|---|---|---|---|---|
| 0.4171 | 99.00 | 94.55-99.97 | 100.00 | 95.07-100.00 |
| 0.5532 | 100.00 | 96.38-100.00 | 100.00 | 95.07-100.00 |

**Spike**

| Cut off | Sensitivity % | CI (%) | Specificity % | CI (%) |
|---|---|---|---|---|
| 0.5010 | 98.02 | 93.03-99.76 | 97.26 | 90.45-99.67 |
| 0.6302 | 99.01 | 94.61-99.97 | 97.26 | 90.45-99.67 |

**Supplementary Figure 2. ROC analysis for RBD and Spike IgG seroconversion detection.** IgG antibody detection in serum samples from SARS-CoV-2 PCR-positive subjects or pre-COVID-19 controls using Immulon 4HBX 96-well plates coated with RBD or Spike proteins as in Figure 3A. Absorbance (Optical density- OD) was evaluated at 450nm at Tecan infinite M200 reader. A) ROC analysis, plotting sensitivity against specificity of RBD (left) or Spike (right) samples as shown in Figure 3A.

## Suppl Figure 3

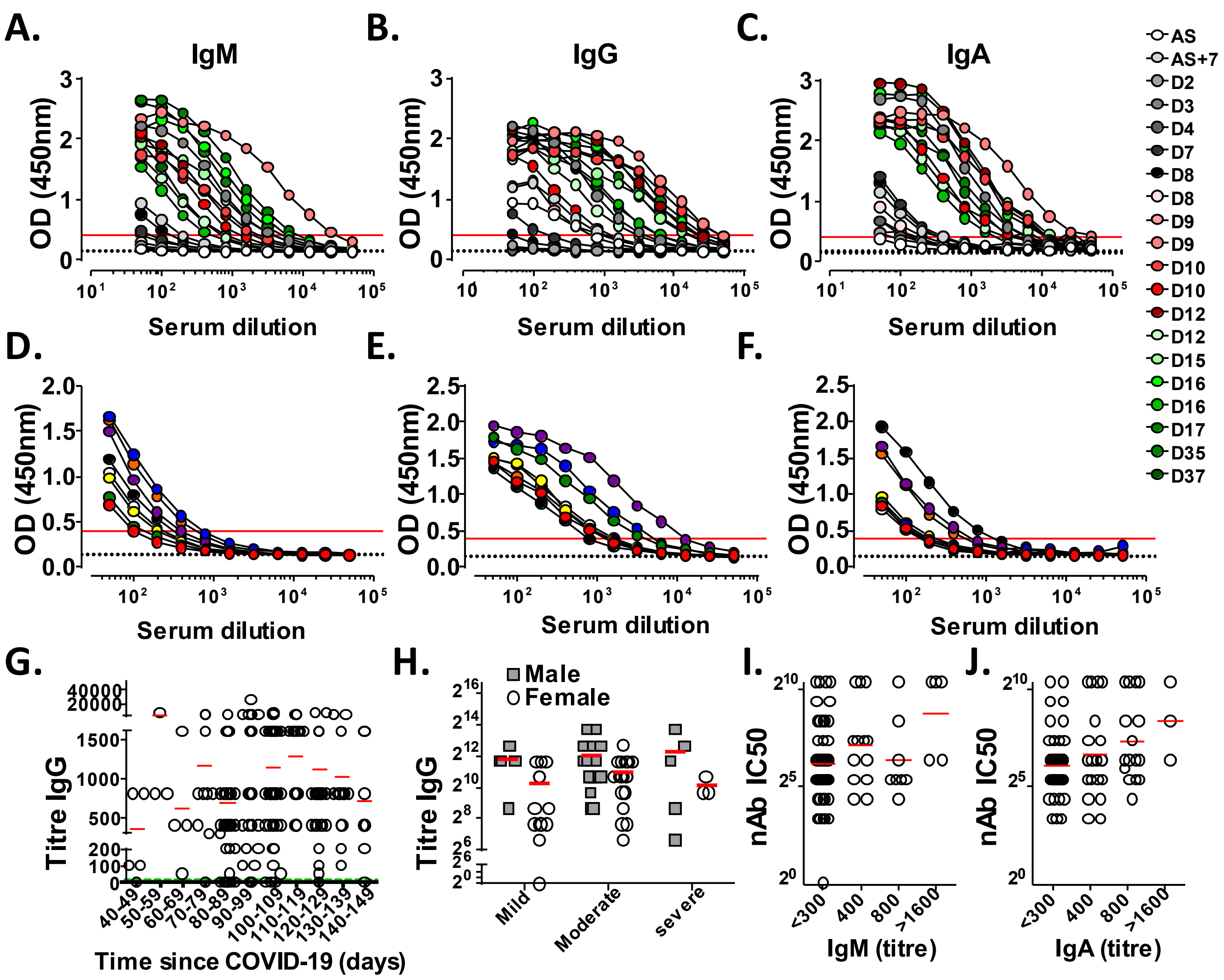


**Supplementary Figure 3. Antibody titre analysis for IgM, IgG and IgA.**

Serum samples from SARS-CoV-2 PCR-positive subjects using Immulon 4HBX 96-well plates coated with RBD protein were serial diluted. Absorbance (Optical density- OD) was evaluated at 450nm at Tecan infinite M200 reader. **A-F**) Antibody titres for A-C) early (<day 40) or D-E) late post COVID19 sera, detecting A,D) IgM, B,E) IgG or C,F) IgA. Legend corresponds to panels A-C, showing the days after COVID19 symptoms or asymptomatic (AS) subjects, or those that were re-sampled seven days later (AS+7). **G**) IgG anti-RBD titre of subjects late (>day 40) after COVID-19 symptoms, with indicated time after SARS-CoV-2 PCR-positive test (n=162). **H**) IgG anti-RBD titre of subjects early (>day 7, < day 40) after COVID-19, experiencing indicated level of symptoms, separated by sex. Two-way ANOVA was used to determine significant differences. No differences were found between disease severity, but differences between male and female was confirmed (P = 0.004). **I-J**) SARS-CoV-2 neutralising activity was determined in sera (n=84) and plotted against I) IgM or J) IgA titre . Red lines indicate geographic mean.

**Materials and methods supplement**

*Materials*

| *Equipment* | *Brand* | *Model/ Cat nº* |
|---|---|---|
| Plate washer | Thermo Scientific | Wellwash 1x8 |
| | Molecular Devices | Aquamax 2000 |
| Plate reader | Tecan | Infinite M200 |
| Manual multichannel pipette (50-250 µL) | VWR | 613-5257P |
| Electronic multichannel pipette (10-300 µL) | VWR | 613-5418 |
| Electronic single channel pipettes | VWR | 613-5425 |
| Pipette controller- Pipetboy | VWR | 612-0928 |
| Reagent reservoir (5bags with 40) | VWR | 613-1183P |
| Sealing Tape for 96-Well Plates | Thermo Scientific | 15036 |

| *Material: ELISA Plates (Surface Treatment)* | *Properties* | *Brand* | *Cat n#* |
|---|---|---|---|
| Immulon 4 HBX | Very Good with Immunoglycoproteins, proteins (water soluble), and good with Gycans. Hydrophobic | Thermo Scientific | 3855 |
| MaxiSorp | Very Good with Immunoglycoproteins, proteins (water soluble), and good with Gycans. Hydrophobic | Merck | M9410-1CS |

| *Reagents: Buffers and antibodies* | *Reagent* | *Brand* | *Cat n#* |
|---|---|---|---|
| Virus inactivation | Triton X-100 | Sigma | T8787-100ml |
| Coating Buffer | PBS 1x (liquid) | Thermo Scientific | A1286301 |
| Blocking buffer | PBS-T + 3% dry milk powder* (w/v) | *Frilabo | sc-2324 |
| Dilution buffer | PBS 1x | | |
| Wash Buffer | PBS 1x + 0.1% (v/v) Tween-20 | | |
| Detergent | Tween-20 | Sigma | P1379 |
| Positive serum | # from confirmed cases of COVID-19 | | |
| Negative serum | #from IMM Biobank (frozen samples) | | |
| Pool of positive serum samples - Quality control (QC) | # from confirmed cases of COVID-19 | | |
| Secondary antibody (enzyme-conjugate)- HPR | Goat Anti-Human IgG+IgM+IgA (HRP) | ab102420 | Abcam |
| | | | Abcam |

| | | | |
|---|---|---|---|
| | Goat Anti-Human IgG Fc (HRP) | ab97225 | |
| | Goat Anti-Human IgM mu chain (HRP) | ab97205 | Abcam |
| | Goat Anti-Human IgA alpha chain (HRP) | ab97215 | Abcam |
| Substrate | TMB Substrate Reagent Set | BD OptEIA™ | 555214 |
| Stop solution | Sulfuric Acid ($H_2SO_4$) | VWR | 85973.290 |

*Plate preparation*

- Recombinant RBD protein
- Recombinant full-length spike protein
- Flat-Bottom Immuno Nonsterile 96-Well Plates 4 HBX (Thermo Scientific #3855)
- Flat Bottom Cell Culture Plates (Corning #3599)
- Triton X-100 (Sigma # T8787-100ml)
- Milk Powder (Frilabo # sc-2324)
- PBS (1x) (Thermo Scientific Gibco # sc-2324)
- Tween 20 (Sigma #P1379)
- TMB Substrate Reagent Set (BD OptEIA™ #555214)
- Sulfuric Acid ($H_2SO_4$) (Sigma #339741)

Secondary antibody (enzyme-conjugate)- HPR

- Goat Anti-Human IgG+IgM+IgA (HRP) (Abcam #ab102420)
- Goat Anti-Human IgG Fc (HRP) (Abcam #ab97225)
- Goat Anti-Human IgM mu chain (HRP) (Abcam #ab97205)
- Goat Anti-Human IgA alpha chain (HRP) (Abcam #ab97215)

Sterile, serological pipettes
■ 5ml (Falcon #356543 or equivalent)
■ 10ml (Falcon #357551 or equivalent)
■ 25 ml (Falcon #357535 or equivalent)

Micropipette tips
■ 10 µl barrier tips (Denville Scientific #P1121 or equivalent)
■ 20 µl barrier tips (Denville Scientific #P1121 or equivalent)
■ 200 µl barrier tips (Denville Scientific #P1122 or equivalent)
■ 200 µl tips (USA Scientific #1111-1700 or equivalent)
■ 1000 µl barrier tips (Denville Scientific #P1126 or equivalent)

Sterile reservoirs (Fisher Scientific #07-200-127 or equivalent)
Micropipettes
Class II biological safety cabinet
Ultra-Low Freezer (-80°C)
Refrigerator at 4°C (+/- 1°C)
Multichannel pipette(s) capable of pipetting 50-250 µl
1.5 ml Eppendorf tubes (Denville #C2170 or equivalent)
Timer

Aquamax 2000 Plate Washer (Molecular Devices #AQUAMAX 2000 or equivalent)
Tecan Infinite M200 microplate Reader or equivalent.
